# Magnetic Phases of Sputter Deposited Thin-Film Erbium

**DOI:** 10.1038/srep39021

**Published:** 2016-12-14

**Authors:** J. D. S. Witt, J. F. K. Cooper, N. Satchell, C. J. Kinane, P. J. Curran, S. J. Bending, S. Langridge, L. J. Heyderman, G. Burnell

**Affiliations:** 1 School of Physics and Astronomy, University of Leeds, Leeds, LS2 9JT, United Kingdom; 2 Laboratory for Mesoscopic Systems, Department of Materials, ETH Zurich, 8093 Zurich, Switzerland; 3 Laboratory for Micro- and Nanotechnology, Paul Scherrer Institute, 5232 Villigen PSI, Switzerland; 4 ISIS, Harwell Science and Innovation Campus, STFC, Oxon OX11 0QX, United Kingdom; 5 Department of Physics, University of Bath, Claverton Down, Bath, BA2 7AY, United Kingdom

## Abstract

We present a detailed structural and magnetic characterization of sputter deposited thin film erbium, determined by x-ray diffraction, transport measurements, magnetometry and neutron diffraction. This provides information on the onset and change of the magnetic state as a function of temperature and applied magnetic field. Many of the features of bulk material are reproduced. Also of interest is the identification of a conical magnetic state which repeats with a wavevector parallel to the c axis *τ*_*c*_ = 4/17 in units of the reciprocal lattice parameter c^*^, which is a state not observed in any other thin film or bulk measurements. The data from the various techniques are combined to construct magnetic field, temperature (*H*, *T*)–phase diagrams for the 200 nm-thick Er sample that serves as a foundation for future exploitation of this complex magnetic thin film system.

With its highly localized 4-*f* electrons and hexagonal close-packed crystal structure, Erbium is an interesting example of a magnetic system where intense competition between the exchange, magnetoelastic and crystal-field energies and their varying dependencies on temperature, strain and applied magnetic field leads to an extremely rich magnetic phase diagram. The understanding of the complex nature of Er and many of the other rare-earth metals and the bulk behaviour of their numerous alloys has lead to many breakthroughs in real-world magnet technology. In this paper we present the characterisation of high-quality thin film sputter deposited Er. The understanding of the effects of reduced dimensionality will assist in the realisation of the many proposed applications requiring complex magnetic thin films. These applications include, for example, the lowering of current densities required in switching spin-transfer-torque (STT) devices^1^ and the nascent fields of spin-triplet superconductivity^2^,^3^ and superconducting spintronics^4^ in which careful control of magnetic non-collinearity is a key ingredient for generating and manipulating the novel effects.

In bulk Er the high-temperature paramagnetic phase undergoes a transition at ~85 K, below which Er possesses three main magnetic regimes. Between 85 and 52 K, the Nèel tempature parallel (*T*_*N*||_) and perpendicular (*T*_*N*⊥_) to the *c*-axis respectively, there exists a long-wavelength sinusoidal antiferromagnet phase confined to the *c*-axis direction with a magnetic repeat distance of approximately 7 atomic layers (magnetic wavevector *τ*_*c*_ = 2/7, in units of the reciprocal lattice parameter, c^*^), known as the *c*-axis modulated (CAM) phase (Fig. 1, right). As the temperature is decreased, this *c*-axis modulation tends from a sine-wave to a square-wave. Below *T*_*N*⊥_ ~ 52 K, in the intermediate phase, the in-plane moments also order (Fig. 1, middle). This ordering has the same magnitude wavevector as the CAM phase upon which it is superimposed. The magnetic repeat distance increases as the temperature decreases, via a number of long-wavelength commensurate phases, to 8 atomic layers (*τ*_*c*_ = 1/4). Finally, below the Curie temperature (*T*_*C*_) ~ 18 K a conical *c*-axis ferromagnetic phase is formed with a wavevector, *τ*_*c*_ = 5/21 (Fig. 1, left). These 3 primary phases were determined initially using neutron diffraction on a bulk Er single-crystal by Cable *et al*.^5^ in the 1960’s.

The exact ordering of the in-plane moments in the intermediate phase remained an open question for some time with several studies indicating the possibility of additional phases therein^6^,^7^,^8^. Over the next few decades, further neutron studies^9^,^10^,^11^,^12^,^13^, the emerging technique of resonant x-ray scattering^14^ and theoretical modelling of this complex system^15^ revealed a multitude of commensurate and incommensurate spin structures within this phase.

For thin film Er, previous work has shown that the magnetic structure is highly sensitive to in-plane strain, with no studies recovering bulk-like behaviour even for films 1 *μ*m thick^16^,^17^. To date, all work has used molecular beam epitaxy (MBE), which produces high-quality films, but is not ideal for incorporation of the thin films in nanoscale devices or in complex heterostructures. In this study we employ magnetron sputtering for its established track record as a growth technique for heterostructures which offers simple commercially scalable routes for devices and we perform structural and magnetic characterisation using X-ray techniques, electrical transport measurements, magnetometry and neutron diffraction to elucidate the complex phase diagram for this system.

## Results and Discussion

### Structural Characterization

In order to understand the magnetic phases of sputter deposited Er, it is necessary to understand the underpinning structure and the ways in which different parameters can modify it. Strain has been demonstrated to influence the magnetic states in a great many systems, and the presence and degree of strain is intimately linked with film thickness and sample temperature. Therefore, the structural characteristics and film quality were investigated using X-ray diffraction (XRD) as a function of these parameters. As described in detail in the Methods section below, a 10 nm Nb seed layer was deposited before growing the Er film in order to provide the necessary crystallographic texture as well as preventing oxidation of the Er as would happen if deposited directly on the Al_2_O_3_ substrate.

**Figure 2:**
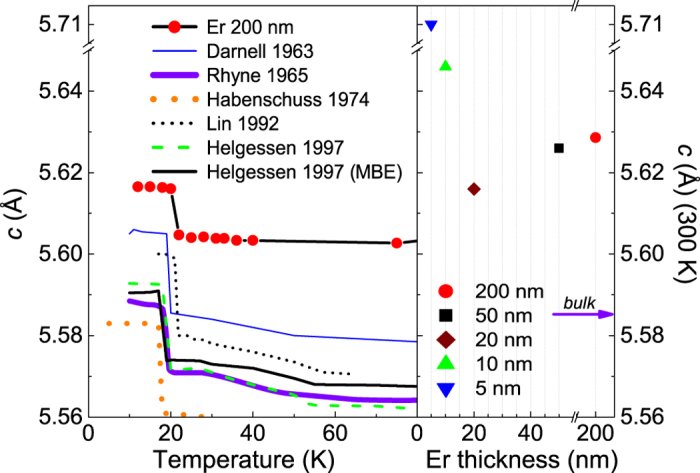
Evolution of the *c*-axis lattice spacing of Er as a function of temperature for the 200 nm-thick sample (left) and as a function of film thickness at 300 K (right). Also shown are the equivalent values for bulk material reproduced from refs 9, 17, 18, 19 and 20 and for an MBE thin film from ref. 17.

The temperature dependence of the Er(0002) peak position for the 200 nm-thick sample was measured in the range 10–300 K, and the calculated *c*-axis lattice spacings are plotted in the left-hand panel of Fig. 2 along with the values for bulk Er over the same temperature range (after refs 9, 17, 18, 19 and 20) and for an MBE thin film (after ref. 17). Firstly, it is apparent that there is a spread in values of the data obtained from the measurements of bulk samples. The measured values for the 200 nm-thick sample are, however, greater than all of these, indicating tensile strain perpendicular to the plane of the sample. The dominance of the Nb(011) and Er(0002) peaks in the high-angle XRD data (not shown) for all but one of the samples (Er 50 nm) indicates a prevailing primary structural phase and that these are highly textured films. This perpendicular to plane strain is therefore predominantly along the *c*-axis, or in the Er[0001] direction. The evolution of the 200 nm-thick Er lattice parameter with temperature is qualitatively similar to that of bulk material, aside from the offset due to strain. The measured strain varies from *ε*_*c*_ = +0.78% at room temperature down to *ε*_*c*_ = +0.51% at 10 K relative to the bulk data from the work of Rhyne and Legvold^19^. In previous work on rare-earth metals grown on Nb buffer material^21^, it has been shown that the Nb contracts smoothly and isotropically with decreasing temperature over this temperature range in good agreement with its (and the sapphire substrate’s) thermal expansion coefficient. In each of the data sets shown in Fig. 2 there is also a clear structural transition at 20–22 K where the lattice parameter abruptly changes. For the 200 nm-thick sample, the change in lattice parameter, Δ*c*/*c* = +0.2%. This is related to the first order transition into the low-temperature ferromagnetic phase. This change is less in the 200 nm-thick sample than that seen in the bulk material, which is the main factor in reducing *ε*_*c*_ at low temperature.

The specific combination of cubic buffer and hexagonal close packed films, presenting the Nb(011) and Er(0002) surface planes, implies that the Er growth has proceeded via the Nishiyama-Wasserman (NW) growth-mode^22^ with the Nb[0
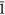
1] direction being parallel to Er[10
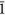
0] direction. This growth-mode requires so-called 3:4 supercell commensuration. The relevant bulk unstrained lattice parameters of the Nb(011) plane presented at the surface are 3.30 Å (Nb[100]) and 4.67 Å (Nb[0
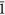
1]) at room temperature. Therefore, if the Nb films were completely relaxed, the Er in-plane strain would be 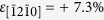 and 
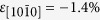
. The more likely scenario, however, is that the Nb films are below their critical thickness and are strained by the Al_2_O_3_ substrate. In-plane XRD was used to determine the crystallographic relationship between the different layers. The Er(0
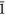
4) planes were found to be offset in *ϕ* by 30 ± 3° from the Al_2_O_3_(0
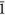
110) planes, indicating that the Al_2_O_3_[01
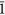
0] direction is parallel to the Er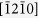 direction. The in-plane Nb peaks were below the resolution of our equipment, but we can infer the relationship between the Al_2_O_3_ and the Nb, knowing the relationships between the Nb and Er and the Al_2_O_3_ and Er. As such, if fully strained, the Nb in-plane strain would be 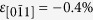 and *ε*_[100]_ = −4.7% and therefore the in-plane Er strain would be 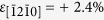 and 
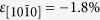
, which seems more reasonable than the rather large 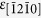 for the fully relaxed case. The crystallographic relationships are shown schematically in Fig 1 in the supplementary information.

The *c*-axis lattice spacings for the differing film thicknesses at room temperature are plotted in the right-hand panel of Fig. 2. All of these are larger than that of the bulk (indicated by the blue arrow). The thinnest Er films, 5 and 10 nm-thick, exhibit the greatest difference from bulk, tending toward the largest *c*-axis lattice spacings. For the thinnest sample measured, the 5 nm-thick sample, the strain is *ε*_*c*_ = +2.24% at room temperature (relative to the bulk data from the work of Rhyne and Legvold^19^). This extension in the *c*-axis direction most likely gives the closest indication of the misfit strain. The lattice spacings for the thicker films, 50 and 200 nm-thick, are both nearer to that of bulk as would be expected due to the relaxation of misfit strain which is possible in thicker films. The trend of lattice spacing with thickness is, however, not monotonic, as would be expected if misfit strain were the only consideration. The lattice parameter of the 20 nm-thick film is the smallest and approaches most closely that of bulk. Similar non-monotonic trends in strain as a function of thickness have been observed for films that undergo an initial Volmer-Weber or 3d-island growth, whereby the strain reaches an extremum at a thickness related to island coalescence. It is possible that these films, which are grown at elevated temperatures, are experiencing modification of the misfit strain due to such an effect.

Using the Scherrer equation and the full widths at half maxima (FWHM) of the *ω*/2*θ* scans, an estimation of the Er crystallite sizes can be made. The calculation gives the shortest crystallite dimension, which in thin films gives an indication of the structural coherence in the direction normal to the sample surface. These values have been calculated from the diffraction data and are given in Table 1. The Nb structural coherence has values ranging from 6.7–7.3 nm indicating that the 10 nm-thick seed layers are consistent between samples and are structurally coherent. The structural coherence for the three thinnest Er films also correspond closely to their thicknesses, indicating high crystallinity throughout the film thickness. In the 50 and 200 nm-thick films, the values for thickness and structural coherence start to diverge. The reason for this in the 50 nm-thick sample is most probably due to an irregularity in the growth temperature, which was most likely lower than the nominal value. This can be inferred from the lack of a distinct sole Nb(011) peak in the X-ray diffraction data. If the growth temperature was lower than expected, the Nb and the Er on top, will be more polycrystalline. The structural coherence of the 200 nm-thick sample is 98.1 nm. This most likely approaches the true crystallite grain size, which is typical of films of this thickness grown in good vacuum conditions^23^, and therefore is likely to reflect the in-plane crystallite size and hence, the in-plane structural coherence for the thinner films.

### Magnetic Characterisation

**Figure 3:**
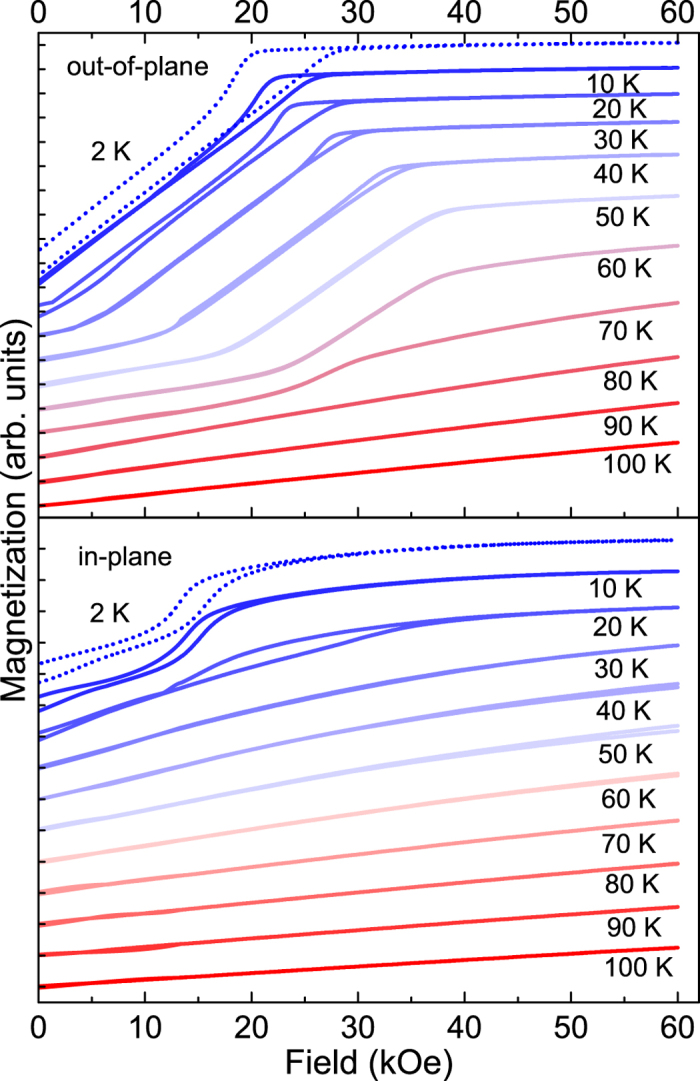
Magnetic hysteresis curves (positive quadrant only) for the 200 nm-thick Er sample for field applied out-of-plane (top) and in-plane (bottom) as a function of temperature. The 2 K curve is shown as a dashed line and all of the curves are offset vertically for clarity with each arbitrary unit tick associated with and corresponding to their respective effective zero magnetization.

The magnetic states of our samples were characterised using SQUID magnetometry. Magnetization as a function of applied magnetic field was measured for various temperatures for the 200 nm-thick sample and these *M*(*H*) loops can be seen in Fig. 3 for magnetic field applied both in-plane (IP) (bottom) and out-of-plane (OOP) (top). The magnetic anisotropy present in the sample can be identified by markedly different shapes of the IP and OOP *M*(*H*) loops. The complexity of the magnetic phase diagram is reflected in the progression of loops which display varying magnetic hysteresis and exhibit a noticeable departure from conventional ferromagnetic or antiferromagnetic *M*(*H*) loops. The key points of note are the difference in transition temperatures from paramagnetism to a magnetically ordered state for the two different orientations. This is identifiable by a shift away from the constant susceptibility associated with paramagnetism. As would be anticipated, the OOP ordering begins between ≈80−90 K whereas the in-plane moments begin to order between ≈50−60 K. Upon ordering, the sample then passes through numerous different states with non-trivial hysteresis at various different points. Ferromagnetism is present below 20 K as evidenced by the finite remanent field.

**Figure 4:**
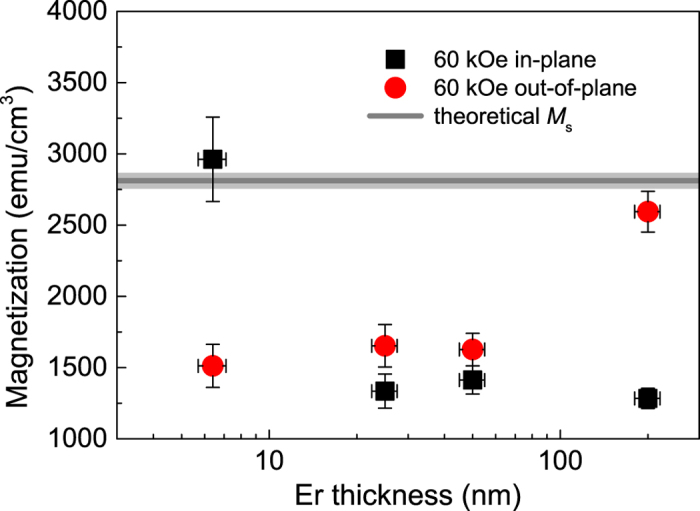
Magnetization at “saturation” in an applied field of 60 kOe at 2 K for in-plane and out-of-plane field orientations as a function of Er thickness. The theoretical maximum is also shown.

The magnetization at an applied field of 60 kOe at 2 K for the different thicknesses of Er is presented in Fig. 4. In bulk material it has been shown that, although the OOP (*c*-axis) magnetization will tend to saturation at relatively small OOP fields, the in-plane components will not reach more than ≈3/5 saturation until field values above ≈100 kOe are applied^12^. The 200 nm-thick sample exhibits the same bulk-like behaviour, with the OOP magnetization approaching the theoretical maximum, ≈2790 emu/cm^3^ (indicated by the grey horizontal line in Fig. 4), whilst the IP magnetization stays at approximately half of this value. For the 50 and 25 nm-thick samples, the IP magnetization remains similar to that of the 200 nm-thick sample, but the OOP magnetization is clearly suppressed. This finite-thickness suppression of the conical phase is common in thin films and suggests that the critical thickness required to retain the bulk-like behaviour is between 50–200 nm.

For the 5 nm-thick sample, the OOP magnetization has a similar value to that of the 25 and 50 nm-thick films, which is consistent with a suppressed conical phase. An important fact to note here is that, relative to the other thicknesses, the magnitudes of the IP and OOP values have switched, with the IP magnetization for this thickness range being much larger than the OOP magnetization and approaching the theoretical maximum. The large increase of the IP magnetization suggests that, as well as a suppression of the conical phase, the long-range ordering is also weakened at this thickness and the film becomes more conventionally ferromagnetic with the magnetization lying in the plane. This change in ordering occurs between an Er thickness of 5–25 nm.

### Neutron Diffraction

Neutron diffraction provides a non-destructive probe of the structural and magnetic repeat distances throughout the entirety of a thin film structure, and therefore the direct determination of any magnetic wavevectors (magnetic repeat distances) that are present. This is of paramount importance when characterising long-wavelength antiferromagnetic structures, such as the spiral phases present in rare-earth materials. Neutron diffraction was carried out on the POLREF reflectometer at the ISIS spallation source.

**Figure 5:**
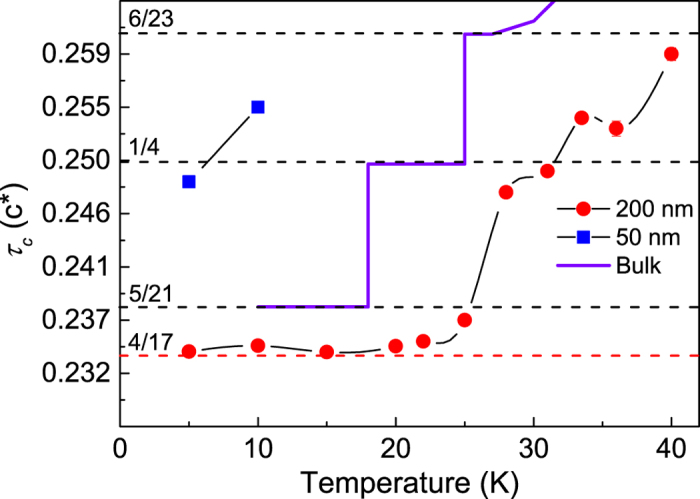
The magnetic wavelength in units of the reciprocal lattice vector as a function of (increasing) temperature for the 200 and 50 nm-thick Er films. The solid black lines are a guide for the eye. Also shown are the values for bulk single-crystal. The dashed black lines are the commensurate bulk phases. The dashed red line indicates the *τ*_*c*_ = 4/17 state.

Temperature dependent neutron diffraction of the (000*τ*_*c*_) peak was performed for the 200, 50 and 5 nm-thick samples, and the data are summarized in Fig. 5. The value of *τ*_*c*_ can be related to the magnetic repeat distance in the *c*-axis direction. When expressed in units of the reciprocal lattice parameter, *τ*_*c*_ = *l*/*n*, where *n* is the number of *c*-axis lattice parameters within which *l* full magnetic repeats are contained (the hcp structure has 2 atomic planes per unit cell). The calculated values are plotted for the 200 and 50 nm-thick Er samples, However, the signal from the 5 nm-thick Er sample was too weak to extract any meaningful results. Also plotted are the values for bulk single-crystal Er and the position of the commensurate phases are indicated. The commensurate phases are points at which the magnetic repeat distance coincides with the structural repeat distance, that is, when *τ*_*c*_ is a rational fraction. Unlike the XRD data shown in Fig. 2, the values for the bulk magnetic repeat distance in this temperature range are very consistent across the literature^9^,^20^ with slight differences at the phase transition between the intermediate and the ferromagnetic phases, *T*_*c*_ ~ 18–20 K manifesting as temperature hysteresis – a difference in the critical ordering temperature depending on whether the sample temperature is increasing or decreasing. In bulk, this hysteresis can be 1–2 K, but for clarity of comparison, only the case where no temperature hysteresis was seen is shown in Fig. 5.

From the data for the 200 nm-thick sample it can be seen that *τ*_*c*_ increases with increasing temperature in a qualitatively similar way to that of bulk. The two most noticeable differences being the general shift to increased temperature and a shift of the lowest temperature state to a lower *τ*_*c*_, or longer magnetic repeat length. From the data for the 50 nm-thick sample it can be seen that at the lowest temperatures the magnetic wavelength approaches that of the commensurate, *τ*_*c*_ = 1/4, state. At higher temperatures, the signal from the 50 nm-thick sample was too weak to measure. The shift to higher temperatures of the data points for the 200 nm-thick sample is not such an unusual phenomenon. As well as a slight shift on increasing temperature in bulk samples, an increased temperature hysteresis is also a common trait in thin films grown by MBE^17^. Something similar to the apparent shift to higher temperatures that can be seen in the data for the 200 nm-thick sample has also been observed before in bulk single-crystal Er upon application of a magnetic field in the *c*-axis direction^20^.

The locking-in at low-temperature to higher values of *τ*_*c*_ in thin films (here 4/17) than is seen in the bulk (2/5) is also not an uncommon occurrence. Indeed, thin Er films to date have not been able to recover the low-temperature *τ*_*c*_ = 5/21 state, reaching at the lowest a *τ*_*c*_ = 6/25(=0.24) state. This is also associated with the suppression of the low-temperature conical ferromagnetic phase. The most intriguing aspect of the data shown in Fig. 5 is the attainment of a hitherto unseen magnetic state. The lowest temperature phase appears to stabilise in a *τ*_*c*_ = 4/17(=0.235) state (indicated in Fig. 5 with a red dashed line), something not seen in any previous measurements and corresponding to a magnetic repeat distance of 8.5 atomic layers in the *c*-axis direction.

### The *H*, *T*–Phase Diagram

**Figure 6:**
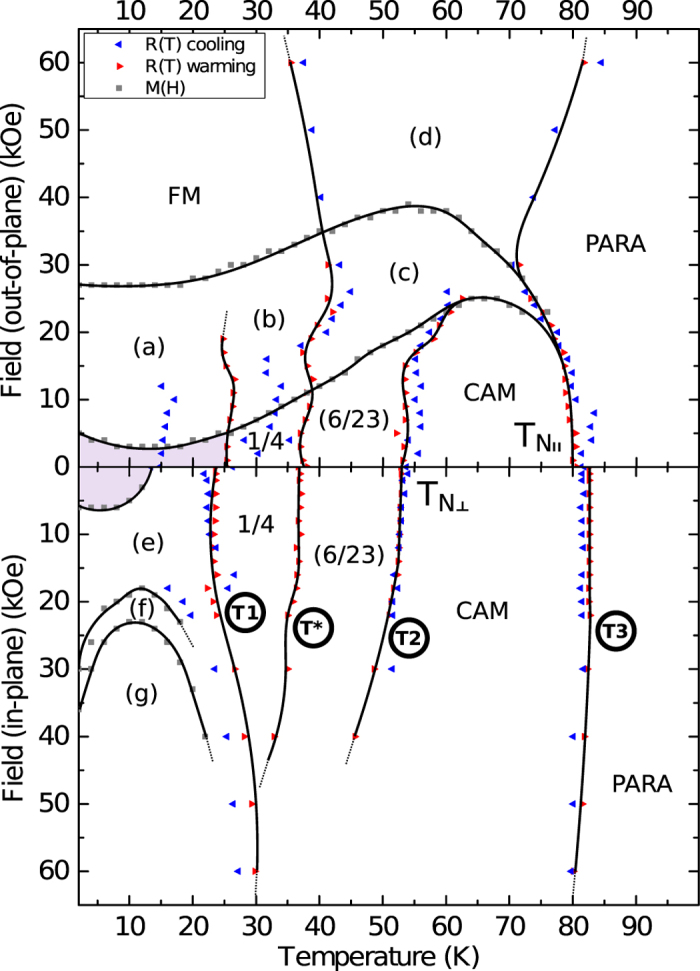
The magnetic phase diagram for the 200 nm-thick Er film showing the magnetic field and temperature parameter space for applied magnetic field out-of-plane (upper panel) and in-plane (lower panel). The boundaries are extracted from resistance and magnetization measurements as a function of applied magnetic field and temperature. Lines and shading are for clarification and the labeling is explained in the text.

In Fig. 6 the magnetic phase diagram for the 200 nm-thick Er sample is presented, which is constructed from the points of change of gradient in resistance versus temperature measurements as a function of applied magnetic field and from the initial magnetization curves (and their numerical derivatives). These points demarcate the various magnetic phase transitions. Lines and shading have been added to the plot for clarity.

Electrical transport measurements were performed using a four-point probe technique in a high-field PPMS system for applied magnetic fields both IP and OOP. Measurements of this type have previously been performed on bulk single-crystal Er^24^,^25^,^26^ and reveal clear links between the points of change in the gradient of the resistance versus temperature plots and changes in the magnetic phase of the system under investigation. In this work the measurements were performed at different constant applied magnetic field values up to 6T. The variations in gradient were recorded for warming and cooling for the 200 nm-thick Er sample only, because the technique was not sensitive enough to detect the changes in the thinner samples. Exemplar resistance versus temperature curves recorded for cooling the 200 nm sample in zero field and in 2 kOe are shown in the supplementary information (Fig. 2). Similarly, exemplar magnetization versus applied field data from Fig. 3 are reproduced in Fig. 3 in the supplementary information to illustrate the identification of phase boundaries in these data.

In the temperature regimes of interest, the resistivity of a sample is dominated by scattering from the population of phonon and magnon states. The number and momentum of excited magnon states, and hence their effect on the resistivity, is dependent not only on the temperature, but also on the energy gap to excite spin-waves and the spin-wave velocity (which in turn depends on the spin-stiffness)^27^. The excitation of spin-waves requires energy to misalign each spin with respect to its local equilibrium position, which in turn is typically determined by the anisotropy energy^28^. Thus in the different magnetic phases the magnon contribution to the overall resistivity would be expected to differ, allowing the phase boundary to be detected in resistance-temperature measurements. The scattering is generally reduced as the magnetic material is cooled (or magnetic field applied) and the moments become more ordered, that is, the energy gap for exciting spin-waves becomes more significant. Also, if the ordered moments possess long-wavelength periodicity, this can introduce so-called superzone boundaries^29^, which are effectively additional prohibited gaps within the first Broullion zone, thus altering the resistance.

At low magnetic fields (points near the central horizontal axis in Fig. 6) the three main magnetic phase boundaries – between the paramagnetic (labeled ‘PARA’) and the CAM phase, between the CAM and the intermediate phase (labeled by the magnetic wavevectors, *τ*_*c*_, measured by neutron diffraction), and between the intermediate and the low-temperature ferromagnetic phase – can be discerned. There is also an additional transition which (with reference to Fig. 5) appears to reflect the transition from the *τ*_*c*_ = 6/23 state to the *τ*_*c*_ = 1/4 state – two long-wavelength commensurate states.

The two lower temperature phase transitions (labeled ‘T1’ and ‘T*’ in Fig. 6) clearly show temperature hysteresis, that is the blue ‘cooling’ and red ‘warming’ symbols do not coincide. This indicates that these transitions are first-order phase transitions. In contrast, the two higher temperature transitions (labeled ‘T2’ and ‘T3’ in the figure), the ‘cooling’ and ‘warming’ data do coincide suggesting these transitions are continuous (or weakly first-order). Somewhat confusingly, the ‘cooling’ data for the IP configuration (lower panel) which would correspond to the ‘warming’ data at ≈36 K (T*) has been pushed down in temperature to coincide with the ≈25 K (T1) warming curve, giving the impression of no hysteresis at this transition, whereas in fact there is. The temperature hysteresis is much more pronounced for the IP configuration. Thermal expansion measurements on bulk single-crystal Er have shown the order of the main phase transitions; paramagnetic to CAM to be continuous, CAM to intermediate to be weakly first-order, and intermediate to ferromagnetic to be first-order. The additional phase transitions within the intermediate phase have either been classed as first-order or the order has not been explicitly determined^24^.

There is great similarity between this phase diagram and the various *H*, *T*–phase diagrams of bulk single-crystal Er measured by neutron diffraction along the *c*-axis^20^,^30^ and *a*, *b*-axes^26^. The states in the 200 nm thick film that have had their magnetic structure specifically determined by neutron diffraction are labelled in Fig. 6 by their magnetic wavevector, *τ*_*c*_ = 1/4 and 6/23. It should be noted that the *τ*_*c*_ = 6/23 state most likely consists of multiple *τ*_*c*_ values, but in the temperature range measured by neutron diffraction, this was the only value recorded. The parentheses here indicate partial state identification. The paramagnetic state was identified using the magnetometry measurements and the CAM phase has been labelled according to its expected phase boundaries, labelled by *T*_*N*||_ and *T*_*N*⊥_. The *τ*_*c*_ = 4/17 phase, identified by neutron diffraction, is indicated by the shaded regions at low fields and low temperature. There has previously been an identification of this wavevector in Er, by Beach *et al*.^31^, but only for the IP modulation (basal plane spiral) and not, as in this work, for the OOP *c*-axis direction.

The remaining phases have been labelled in accordance with literature^20^,^26^,^30^. The (a) phase is most likely associated with the conical, *τ*_*c*_ = 5/21 or 1/4 phases. The ferromagnetic phase, ‘FM’, in the low temperature, high field region of the OOP plot was determined from the hysteresis loops presented in Fig. 3 and the theoretical saturation values presented in Fig. 4. Watson and Ali^32^ identified a phase boundary similar to that between (c) and (d) (in their nomenclature phases III and II, respectively), which have previously been classified as incommensurate or ferromagnetic phases, but the exact structure of both of these phases remains unknown. From the work of Frazer *et al*.^26^ the (e) to (f) to (g) transitions appear to correspond to the progression from ‘cone’, (*τ*_*c*_ = 5/21), through ‘fan’ (*τ*_*c*_ = 1/4), to ferromagnetic ‘canted fan’ phase.

## Summary and Conclusion

In this work we have shown that many of the features of bulk Er are attainable in thin film form. However, as the Er thickness is decreased the characteristic features, such as a low-temperature ferromagnetic OOP conical magnetism, are weakened. The 200 nm-thick film was found to retain all of the key features of bulk single-crystal Er, possessing the three main magnetic regimes. The key difference in its behaviour compared to that of bulk was that the 200 nm-thick film reached a low-temperature magnetic phase of longer magnetic repeat distance than has been reported elsewhere, which was shown using neutron diffraction. We attribute the origin of this phase to strain in the thin films, which was revealed by temperature dependent XRD, that placed the *c*-axis lattice constants at values greater than any reported elsewhere.

The thinner films showed varying suppression of the low-temperature magnetic phases. For the 50 and 25 nm-thick samples, it was shown with magnetometry measurements that, at these thicknesses, the OOP magnetism had been reduced, suggesting a suppression of the transition into the low-temperature conical phase. This suppression of an OOP component of magnetism most likely arises because the contribution of the demagnetizing energy begins to outweigh that from the magnetocrystalline anisotropy, typical of films of decreasing thickness. The thinnest, 5 nm-thick Er film, exhibited signs of suppression of the conical phase and a weakening of the spiral phase. This was identified by the fact that the IP magnetization approached the theoretical maximum at 60 kOem which was not the case for the thicker film at this applied field. This is because the stability of the spiral is based upon a long range ordering effect with the stability lessened toward the spiral ends. As the film thickness is decreased, the ends of the spiral begin to constitute a more significant fraction of the whole, which is essentially a surface area to volume ratio effect. The spiral ends, having a lower coordination and therefore lessened exchange stabilisation, effectively act as a handle with which the applied field can unzip the spiral.

In conclusion, we have shown that high-quality thin film Er can be grown by sputter deposition and that bulk-like behaviour can be retained in films of 200 nm thickness. For films thinner than 200 nm, the growth induced strain becomes increasingly important which has implications for thin film incorporation into device structures, particularly with the advance of areas of device research which require nanoscale control over magnetic inhomogeneity. We have also shown that the various rich magnetic phases, which can be seen in bulk material, are also observable in thin films and, using a combination of techniques, we were able to construct a magnetic field, temperature (*H*, *T*)–phase diagram. The results of this work will serve as a valuable aid for incorporating specific magnetic non-homogeneity into Er-based thin film devices structures.

## Methods

The samples were prepared by DC magnetron sputtering onto *c*-plane Al_2_O_3_ substrates in a system with a base pressure of 10^−7^ mbar in a single vacuum cycle. Samples considered in this study have a Nb seed layer with a thickness of 10 nm, the Er film with nominal thickness ranging from 5–200 nm and a Lu capping layer with a thickness of 5 nm. Lu weas used as a cap due to its excellent lattice matching with Er which minimizes strain in the upper layers of the Er films, In addition, being a refractory metal Lu could be grown as a smooth layer on a heated substrate unlike traditional capping materials such as Au. The best growth was achieved with the Nb seed layer deposited at a nominal temperature of 700 °C before cooling to a nominal temperature of 500 °C for the Er and Lu depositions. Growth proceeded at a typical Ar flow of 55 sccm and pressure of 2–3 mbar, a substrate-sample distance of approximately 25 mm and at a typical growth rate of 0.1 nm s^−1^. To ensure consistency between samples, up to 24 complete samples were deposited in a single vacuum cycle. Growth rates for each material were calibrated using fits to the Kiessig fringes in X-ray reflectivity measurements.

### Availability of Data

The data associated with this paper are openly available from the University of Leeds data repository, http://doi.org/10.5518/112.

## Additional Information

**How to cite this article**: Witt, J. D. S. *et al*. Magnetic Phases of Sputter Deposited Thin-Film Erbium. *Sci. Rep.* **6**, 39021; doi: 10.1038/srep39021 (2016).

**Publisher's note:** Springer Nature remains neutral with regard to jurisdictional claims in published maps and institutional affiliations.

## Supplementary Material

Supplementary Information

## Figures and Tables

**Figure 1:**
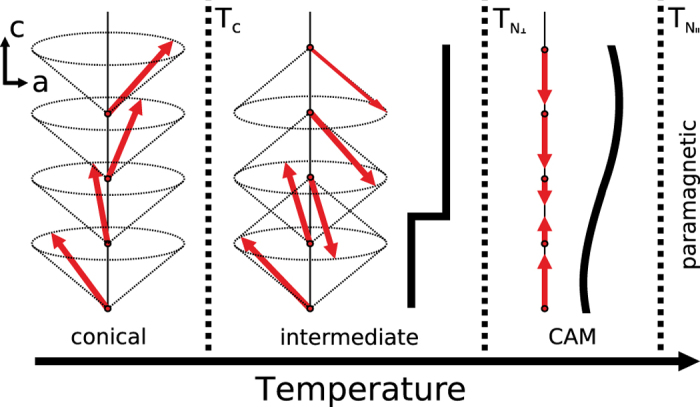
Schematic showing the magnetic structure as a function of temperature for bulk Er. The heavy black lines beside the intermediate and CAM phases indicate the ‘squaring-up’ of the *c*-axis modulation with decreasing temperature.

**Table 1:** The FWHM for the *ω*/2*θ* scans and the calculated structural coherence.

Er thickness	FWHM (°)		Structural coherence	
(nm)	Nb(011)	Er(0002)	Nb (nm)	Er (nm)
200	1.15	0.09	7.3	91.8
50	*	0.28	—	29.4
20	1.15	0.44	7.3	18.8
10	1.26	0.95	6.7	8.7
5	1.16	1.52	7.3	5.4

^*^The Nb structural phase was clearly different in the 50 nm thick sample than the others possibly due to a reduced heater temperature. This also led to a reduced Er structural coherence and a mixed Er phase with a strong secondary peak of Er(011) with a comparable structural coherence of 29.5 nm.
