# Supplementary material for: Magnetic Phases of Sputter Deposited Thin-Film Erbium
Source: Sci Rep. 2016 Dec 14;6:39021. doi: 10.1038/srep39021 (PMC5155211; doi:10.1038/srep39021)
Supplement: Supplementary Information [file srep39021-s1.pdf]

Magnetic Phases of Sputter Deposited Thin-Film Erbium

J. D. S. Witt[1,2,3], J. F. K. Cooper[4], N. Satchell[1,4], C. J. Kinane[4], P. J. Curran[5], S. J. Bending[5], S. Langridge[4], L. J. Heyderman[2,3], and G. Burnell[1,*]

[1]School of Physics and Astronomy, University of Leeds, Leeds, LS2 9JT, United Kingdom
[2]Laboratory for Mesoscopic Systems, Department of Materials, ETH Zurich, 8093 Zurich, Switzerland
[3]Laboratory for Micro- and Nanotechnology, Paul Scherrer Institute, 5232 Villigen PSI, Switzerland
[4]ISIS, Harwell Science and Innovation Campus, STFC, Oxon OX11 0QX, United Kingdom
[5]Department of Physics, University of Bath, Claverton Down, Bath, BA2 7AY, United Kingdom
[*]Corresponding Author

ABSTRACT

Supplementary Information

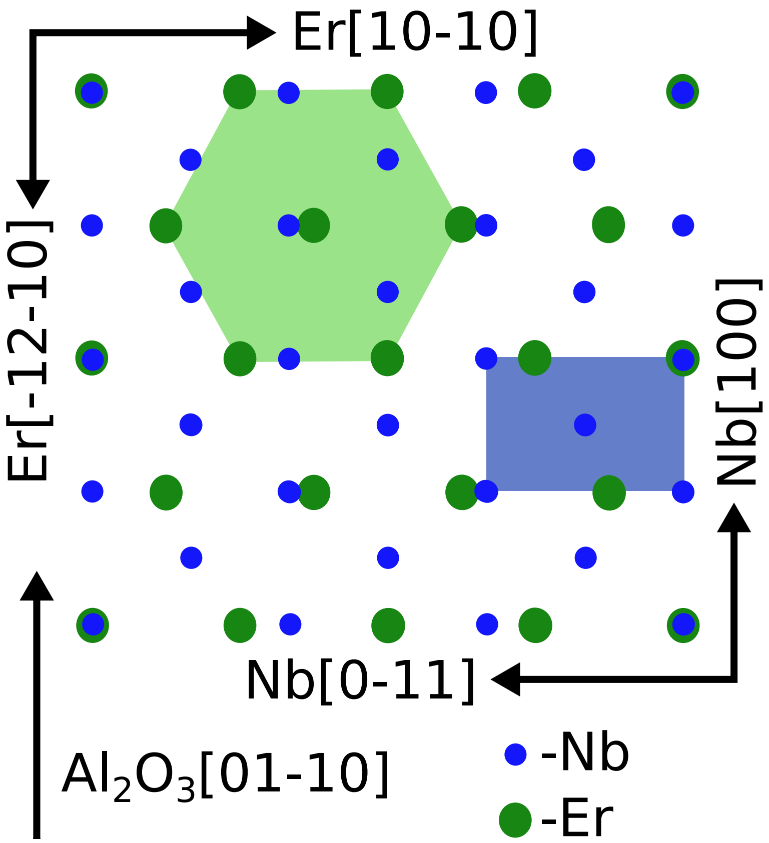

Figure 1. A schematic showing the crystallographic relationships between the Al_2O_3 substrate and the Nb and Er films. The $Al_2O_3[01\bar{1}0]$ direction is parallel to the Er $[\bar{1}2\bar{1}0]$ direction, and the $Al_2O_3[01\bar{1}0]$ direction is parallel to Nb $[100]$, giving Er $[10\bar{1}0]$ prallel to Nb $[0\bar{1}1]$.

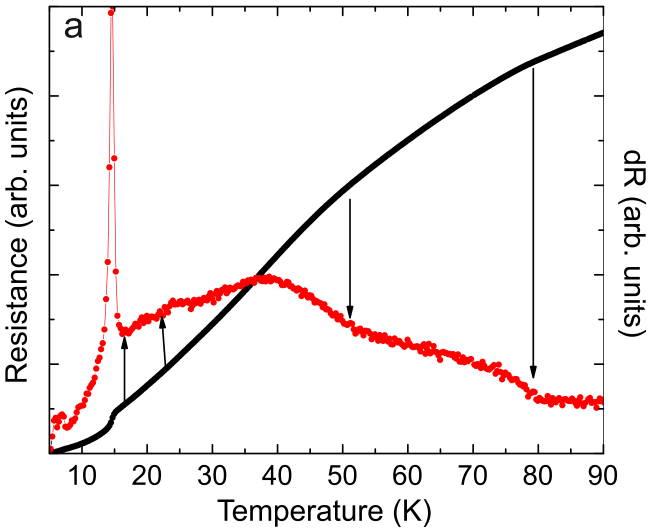

Figure 2. Exemplar resistance versus temperature data as used to construct the phase diagram shown in figure 6 of the main text. (a) Data taken at zero applied field and during cooling and (b) data taken at an applied field of 2 kOe out of plane, also during cooling. As a guide to the eye for identifying the phase boundaries the data are also shown differentiated with respect to temperature *red curves) and as resistance (black curves). The vertical arrows indicate the positions of the phase boundaries, determined as points of inflection in the resistance data.

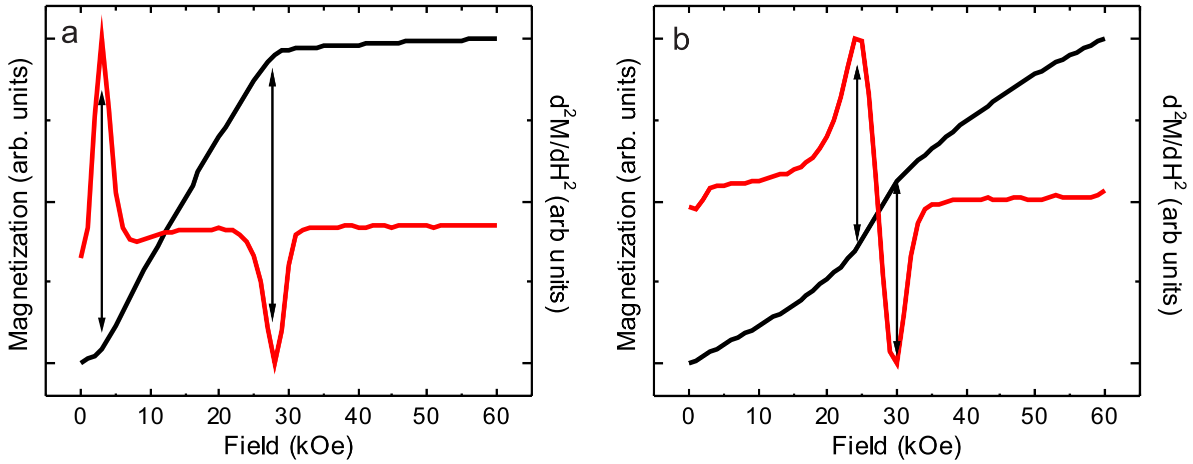

Figure 3. Exemplar magnetization versus out-of-plane applied field data reproduced from figure 3 in the main text as used to construct the phase diagram shown in figure 6 of the main text. Data taken at (a) 10 K and (b) 70 K for the zero to positive applied magnetic field quadrant of the hysteresis loop (black curves). As a guide to the eye for identifying the phase boundaries the data are also shown as the second differential with respect to applied magnetic field *red curves. The vertical arrows indicate the positions of the phase boundaries, determined as points of inflection in the magnetization data.
